# *FYCO1* Frameshift Deletion in Wirehaired Pointing Griffon Dogs with Juvenile Cataract

**DOI:** 10.3390/genes13020334

**Published:** 2022-02-11

**Authors:** Gabriela Rudd Garces, Matthias Christen, Robert Loechel, Vidhya Jagannathan, Tosso Leeb

**Affiliations:** 1 Institute of Genetics, Vetsuisse Faculty, University of Bern, 3001 Bern, Switzerland; gabriela.ruddgarces@vetsuisse.unibe.ch (G.R.G.); matthias.christen@vetsuisse.unibe.ch (M.C.); vidhya.jagannathan@vetsuisse.unibe.ch (V.J.); 2 Institute of Veterinary Genetics “Ing. Fernando Noel Dulout”, National University of La Plata, La Plata 1900, Argentina; 3 VetGen, Ann Arbor, MI 48108, USA; loechel@umich.edu

**Keywords:** *Canis lupus familiaris*, whole-genome sequence, ophthalmology, lens, animal model, precision medicine, veterinary medicine

## Abstract

Different breed-specific inherited cataracts have been described in dogs. In this study, we investigated an inbred family of Wirehaired Pointing Griffon dogs in which three offspring were affected by juvenile cataract. The pedigree suggested monogenic autosomal recessive inheritance of the trait. Whole-genome sequencing of an affected dog revealed 12 protein-changing variants that were not present in 566 control genomes, of which two were located in functional candidate genes, *FYCO1* and *CRYGB*. Targeted genotyping of both variants in the investigated family excluded *CRYGB* and revealed perfect co-segregation of the *FYCO1* variant with the juvenile cataract phenotype. This variant, *FYCO1*:c.2024delG, represents a 1 bp frameshift deletion predicted to truncate ~50% of the open reading frame p.(Ser675Thrfs*5). *FYCO1* encodes the FYVE and coiled-coil domain autophagy adaptor 1, a known regulator of lens autophagy, which is required for the normal homeostasis in the eye. In humans, at least 37 pathogenic variants in *FYCO1* have been shown to cause autosomal recessive cataract. *Fcyo1^−/−^* knockout mice also develop cataracts. Together with the current knowledge on *FYCO1* variants and their functional impact in humans and mice, our data strongly suggest *FYCO1*:c.2024delG as a candidate causative variant for the observed juvenile cataract in Wirehaired Pointing Griffon dogs. To the best of our knowledge, this study represents the first report of a *FYCO1*-related cataract in domestic animals.

## 1. Introduction

A cataract refers to opacity or light scattering in the lens of the eye leading to visual impairment or blindness [1]. It is caused by alterations of lens homeostasis due to accumulation of high-molecular-weight protein aggregations or a disarray in the organization of lens fiber cells due to aberrations during development or growth [1]. In human medicine, a cataract is classified as age-related, congenital, or secondary to other causes. Congenital cataracts are diagnosed within the first year of life, and up to 25% of cases are hereditary [1,2]. Inherited forms of cataract are clinically heterogeneous with syndromic and non-syndromic phenotypes [3,4]. Variants in more than 30 genes have been shown to cause different forms of cataracts in humans [5,6]. These genes encode structural components of the lens or proteins involved in the homeostasis of the lens such as crystallins, transcription factors, membrane gap junction proteins, chaperones, or cytoskeletal proteins [5,6,7].

In dogs, cataracts are one of the most frequent ocular diseases and represent a leading cause of blindness [8]. Several breed-specific cataracts have been described without identifying the underlying causative genetic defects [9,10,11,12,13]. So far, variants in only one gene have been identified in canine cataracts. *HSF4* encoding heat-shock transcription factor 4, was found to be mutated in affected dogs from three different breeds (OMIA 001758-9615) [14]. A homozygous single-base insertion in exon 9 of *HSF4* causes primary bilateral cataract in Staffordshire Bull Terriers and Boston Terriers [14,15]. A single-base deletion is associated with dominant bilateral posterior polar subcapsular cataract in Australian Shepherds [14,16]. However, as some of the affected Australian Shepherds were clear of the mutant *HSF4* allele, additional susceptibility loci were postulated [16]. A subsequent genome-wide association study in *HSF4^+/+^* controls and cases indicated a 14.16 Mb region on chromosome 13 associated with the observed phenotype. However, no causal variant or obvious candidate genes were found within the critical interval [17].

No association with *HSF4* variants was found in juvenile cataract-affected English Cocker Spaniels, Kromfohrländers, Dachshunds, Entlebucher Mountain dogs, or Jack Russell Terriers [9,18,19]. These results suggest that additional pathogenic variants lead to different forms of canine hereditary cataracts.

The present study was initiated after a breeder reported puppies with juvenile cataracts in several related litters of Wirehaired Pointing Griffon dogs. The aim of our study was to unravel the causative genetic defect.

## 2. Materials and Methods

### 2.1. Animals Selection for Genetic Analyses

This study included 24 related Wirehaired Pointing Griffon dogs originating in Europe. They comprised three affected littermates with juvenile cataract, their unaffected parents, and 19 unaffected relatives. Phenotypes were reported by the owners. We additionally used samples from 32 unrelated Wirehaired Pointing Griffon dogs originating in North America.

### 2.2. DNA Isolation

EDTA blood samples were collected for genomic DNA isolation. Genomic DNA was isolated from 500 µL of EDTA blood with the Maxwell RSC Whole-Blood Kit using a Maxwell RSC instrument (Promega, Dübendorf, Switzerland).

### 2.3. Whole-Genome Sequencing of an Affected Dog

An Illumina TruSeq PCR-free DNA library with ~400 bp insert size was prepared from genomic DNA of an affected Wirehaired Pointing Griffon dog. We collected 225 million 150 bp paired-end reads on a NovaSeq 6000 instrument (25× coverage). The reads were mapped to the UU_Cfam_GSD_1.0 dog reference genome assembly as previously described [20]. The sequence data were deposited under study accession PRJEB16012 and sample accession SAMEA10644717 at the European Nucleotide Archive.

### 2.4. Variant Calling and Filtering

Variant calling in a set of 567 dog genome sequences representing 148 different dog breeds was performed as described [20]. The accession numbers of all genome sequences are compiled in Supplementary Table S1. To predict the functional effects of the called variants, the SnpEff software [21], together with NCBI annotation release 106 for the UU_Cfam_GSD_1.0 genome reference assembly, was used. For variant filtering, we used the genome of the affected Wirehaired Pointing Griffon and 566 dogs from other breeds that were assumed to be clear of the pathogenic allele. We employed a hard filtering approach to identify variants for which the affected dog was homozygous for the alternate allele (1/1) while the 566 control genomes were either homozygous for the reference allele (0/0) or had a missing genotype call (/). Short read alignments of the sequencing data were visualized with the Integrative Genomics Viewer (IGV) [22].

Private variants in the affected dog were prioritized according to functional knowledge. We concentrated on variants predicted to change an encoded protein (SnpEff impact: high or moderate). Further prioritization was performed on the basis of searches in online databases (OMIA [23], OMIM [24], MGI [25]) and the scientific literature.

### 2.5. Gene Analysis

The UU_Cfam_GSD_1.0 dog reference genome assembly and the NCBI annotation release 106 were used. Numbering within the canine *FYCO1* gene corresponds to the NCBI RefSeq accession numbers XM_038566669.1 (mRNA) and XP_038422597.1 (protein). Numbering within the canine *CRYGB* gene corresponds to NM_001110799.1 (mRNA) and NP_001104269.1 (protein).

### 2.6. Targeted Genotyping and Sanger Sequencing

The *FYCO1*:c.2024del variant was genotyped by direct Sanger sequencing of PCR amplicons. A 327 bp PCR product was amplified from genomic DNA using AmpliTaqGold360Mastermix (Thermo Fisher Scientific, Waltham, MA, USA) together with primers 5′–CTGATTACCAGGCCCTGCAG–3′ (Primer F) and 5′–GGCCTTTCTCTGTCGTGAGG–3′ (Primer R). PCR was performed with an initial long denaturation of 10 min at 95 °C, followed by 35 cycles of 30 s denaturation at 95 °C, 30 s annealing at 60 °C, and 60 s polymerization at 72 °C. A final extension of 7 min at 72 °C was performed. A 5200 Fragment Analyzer was used for the quality control of PCR products (Agilent, Santa Clara, CA, USA). After treatment with shrimp alkaline phosphatase and exonuclease I, PCR amplicons were sequenced on an ABI 3730 DNA Analyzer (Thermo Fisher Scientific, Waltham, MA, USA) The sequencing reaction was performed with an initial long denaturation of 1 min at 96 °C, followed by 25 cycles of 10 s denaturation at 96 °C, 5 s annealing at 50 °C, and 2 min polymerization at 60 °C. Sanger sequences were analyzed using the Sequencher 5.1 software (GeneCodes, Ann Arbor, MI, USA). The *CRYGB*:c.367C>T variant was genotyped with the same methodology and the following primers for the PCR amplification: 5′–GGCACCCCAGTCAAGGTATC–3′ (Primer F) and 5′–TTCTCTCTGCCTCTCCAGCA–3′ (Primer R).

### 2.7. In Silico Predictions

PROVEAN [26], MutPred2 [27], and PredictSNP1 [28] were used to predict the functional consequences of the *CRYGB*:p.(His123Tyr) missense variant on the γ-crystallin B protein. PROVEAN scores of less than −2.5 and MutPred2 scores of more than 0.80 were considered deleterious predictions. PredictSNP1 combines the outputs of six different software tools into a composite score that represents an estimated probability of the accuracy of the prediction (deleterious or neutral).

## 3. Results

A breeder noticed signs of body imbalance in the affected puppies and difficulties following the other littermates at 8 weeks of age. Private clinical and ophthalmological examinations revealed the presence of opaque spots in the eyes and blindness, corresponding to signs of juvenile cataract. This prompted the beginning of a genetic investigation.

**Figure 1:**
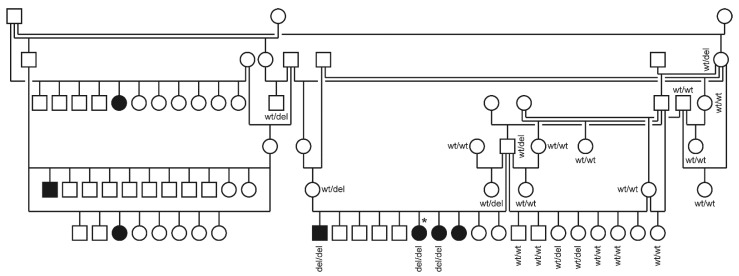
Pedigree of the investigated Wirehaired Pointing Griffon family. All dogs of this pedigree originated in Europe. Filled symbols indicate affected dogs and open symbols indicate nonaffected dogs. Squares and circles represent males and females, respectively. An asterisk indicates the dog that was used for whole-genome sequencing. Genotypes at the *FYCO1*:c.2024del variant are given for dogs from which samples were available.

We obtained blood samples from three affected littermate puppies and their unaffected parents. The sire and dam had reportedly produced offspring affected by juvenile cataract in previous litters. However, samples from these dogs were not available. The pedigree showed multiple inbreeding loops and was suggestive for a monogenic autosomal recessive mode of inheritance of the trait (Figure 1).

The genome of one affected dog was sequenced at ~25× coverage, and variants were called with respect to the UU_Cfam_GSD_1.0 reference genome assembly. Subsequently, we searched for private homozygous variants in the genome of the affected dog that were not present in 566 control genomes. The variant calling pipeline detected 12 private protein-changing variants in 12 different genes (Table 1, Supplementary Table S2).

Prioritization of private variants according to functional knowledge of the affected genes revealed two candidate variants for the observed juvenile cataract. The other 10 variants affected genes not known to be involved in eye development or homeostasis. The first of these two plausible candidates represented a missense variant in the *CRYGB* gene encoding the γ-crystallin B protein. This missense variant, NP_001104269.1:p.(His123Tyr), was predicted to have a neutral impact on the protein product (PROVEAN score: −2.101; MutPred2 score: 0.614; PredictSNP1 score: 83%). Targeted genotyping of all available family members did not show co-segregation with the phenotype as it revealed an affected dog that was homozygous for the wildtype allele.

**Figure 2:**
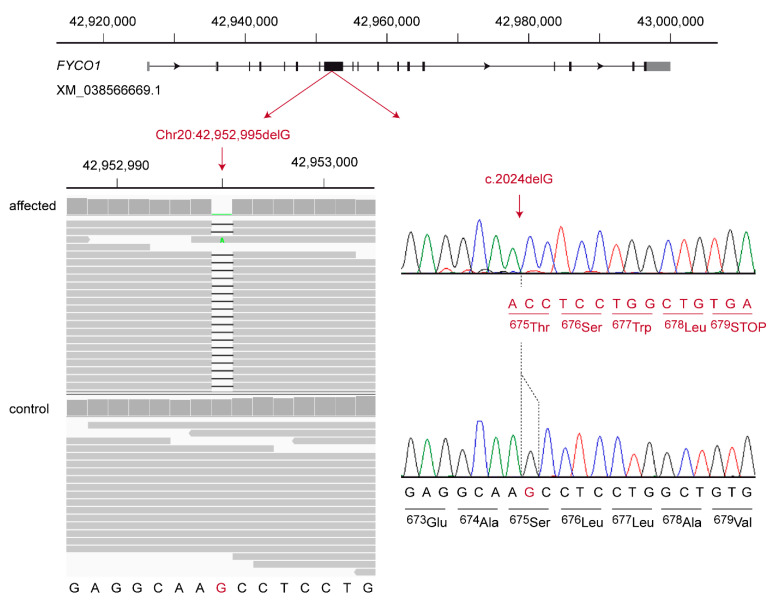
Details of the *FYCO1*:c.2024delG variant. The genomic organization of the *FYCO1* gene on chromosome 20 is indicated at the top. At the bottom left, an Integrative Genomics Viewer (IGV) screenshot shows the short-read alignments of the affected and a control dog at the position of the deletion. At the bottom right, representative Sanger electropherograms of an affected and a control dog are shown. The altered reading frame and the premature stop codon of the mutant sequence are indicated in red. More than 50% of the 1459 wildtype codons are truncated.

The second candidate variant was a 1 bp frameshift deletion in the *FYCO1* gene encoding the FYVE and coiled-coil domain autophagy adaptor 1. It can be designated as XM_038566669.1:c.2024delG or Chr20:42,952,995del (UU_Cfam_GSD_1.0) (Figure 2). This frameshift deletion introduces a premature stop codon and is predicted to truncate ~50% of the open reading frame, XP_038422597.1:p.(Ser675Thrfs*5).

We determined the genotypes at *FYCO1*:c.2024delG and *CRYGB*:c.367C>T in a cohort comprising 56 Wirehaired Pointing Griffon dogs, including the index family. Genotypes at the *CRYGB*:c.367C>T variant did not show perfect genotype–phenotype association, and the alternative allele was present at 17% frequency in 32 unrelated Wirehaired Pointing Griffon dogs from North America (Table 2). In contrast, the genotypes at *FYCO1*:c.2024delG showed perfect association and co-segregation with the phenotype in the family. The three affected littermates were homozygous for the *FYCO1* variant while the parents were heterozygous carriers. The remaining 19 unaffected relatives were either heterozygous or homozygous for the wildtype allele of *FYCO1* (Figure 1). The mutant allele was not observed in a small cohort of 32 unrelated Wirehaired Pointing Griffon dogs from North America (Table 2).

## 4. Discussion

In this study, we investigated a new form of juvenile cataract in Wirehaired Pointing Griffon dogs. The pedigree analysis suggested an autosomal recessive mode of inheritance. The molecular genetic analysis of the trait identified a homozygous frameshift deletion in the *FYCO1* gene in three available affected puppies. While we found the mutant allele only in the affected dogs and some close relatives, our study was limited to a very small number of dogs that cannot be considered representative for the breed. It is therefore not clear how far the genetic defect has already spread.

We have to caution that our variant discovery methodology was limited to the search for small variants, involving up to approximately 20 nucleotides at most. As this approach yielded a very plausible candidate causal variant, we did not further investigate potential large structural variants. The possibility of a structural variant causing the juvenile cataracts cannot be formally excluded. We also based our search on the hypothesis that the disease-causing variant is unique to Wirehaired Pointing Griffon dogs and not present in any of the 566 control genomes.

*FYCO1* encodes the FYVE and coiled-coil domain autophagy adaptor 1, which is conserved in vertebrates and widely expressed, especially in the heart, skeletal muscle, and eye [29,30]. The FYCO1 protein is a phosphatidylinositol 3-phosphate binding protein and mediates microtubule plus-end-directed vesicle transport. It is localized on the external membrane of autophagosomes and binds to the microtubule-associated protein 1 light chain 3 (LC3), the mammalian homolog of the yeast Atg8 protein [31,32]. In the lens, autophagy plays a key role in fiber cell maturation and formation of the organelle-free zone, which is essential to lens transparency [33,34].

Disruption of the gene in *Fyco1*^−/−^ knockout mice resulted in impaired autophagy, decreased conversion of LC3-I to LC3-II, and accumulation of p62. Furthermore, in the eyes of *Fyco1*^−/−^ knockout mice, FYCO1-mediated recruitment of damaged α-crystallin A and B into autophagosomes was disrupted, which led to an accumulation of degenerated α-crystallin in the lens and cataract formation [32].

In humans, *FYCO1* loss-of-function variants were reported to cause autosomal recessive cataract 18 (OMIM 607182) [35,36,37,38,39]. Affected individuals had bilateral nuclear cataracts that were either present at birth or developed in infancy. No other ocular or systemic abnormalities were present in the investigated patients [35]. At least 37 different pathogenic *FYCO1* variants were identified in patients with cataracts [39].

The *FYCO1*:c.2024delG frameshift deletion identified in the affected Wirehaired Pointing Griffon dog of this study leads to a premature stop codon. We consider it therefore unlikely that any functional FYCO1 protein is expressed in homozygous mutant dogs. Unfortunately, an experimental confirmation on the transcript or protein level could not be performed, as no suitable tissue samples were available. Considering the comprehensive knowledge on *FYCO1* function in humans and mice, together with the observed genotype–phenotype co-segregation in the index family, we think that *FYCO1*:c.2024delG represents a very likely candidate causative variant for juvenile cataract in the investigated Wirehaired Pointing Griffon dogs.

## 5. Conclusions

In summary, we revealed a new autosomal recessive cataract in Wirehaired Pointing Griffon dogs and identified the *FYCO1*:c.2024delG frameshift deletion as a candidate causative variant. Our data enable genetic testing to prevent the unintentional breeding of affected puppies and provide a potential spontaneous large animal model for *FYCO1*-related cataract.

## Figures and Tables

**Table 1:** Homozygous variants detected by whole-genome resequencing of an affected dog.

Filtering Step	Variants
Variants in whole genome	2,626,490
Private variants (absent from 566 control genomes)	1965
Protein-changing private variants	12
Protein-changing private variants in functional candidate genes	2

**Table 2:** Association of the genotypes at two candidate variants with juvenile cataract.

Phenotype	*FYCO1*:c.2024delG			*CRYGB*:c.367C>T		
	*ref/ref*	*ref/alt*	*alt/alt*	*ref/ref*	*ref/alt*	*alt/alt*
Cases (*n* = 3)	-	-	3	1	-	2
Nonaffected parents (obligate carriers, *n* = 2)	-	2	-	-	2	-
Nonaffected relatives (*n* = 19)	13	6	-	9	10	-
Unrelated Wirehaired Pointing Griffon dogs (*n* = 32)	32	-	-	21	11	-

## Data Availability

The genome sequence data used in this study are available from the European Nucleotide Archive. Accession numbers are given in Table S1.

## Supplementary Materials

The following supporting information can be downloaded at https://www.mdpi.com/article/10.3390/genes13020334/s1: Table S1. Accession numbers of 567 dog genome sequences; Table S2. Private variants in the genome of the sequenced affected Wirehaired Pointing Griffon dog.
